# PqsA Promotes Pyoverdine Production via Biofilm Formation

**DOI:** 10.3390/pathogens7010003

**Published:** 2017-12-25

**Authors:** Donghoon Kang, Kelly E. Turner, Natalia V. Kirienko

**Affiliations:** Department of Biosciences, Rice University, Houston, TX 77005, USA; dk30@rice.edu (D.K.); ket2@rice.edu (K.E.T.)

**Keywords:** *Pseudomonas aeruginosa*, PQS signaling, biofilm, cell aggregation, pyoverdine, virulence

## Abstract

Biofilms create an impermeable barrier against antimicrobial treatment and immune cell access, severely complicating treatment and clearance of nosocomial *Pseudomonas aeruginosa* infections. We recently reported that biofilm also contributes to pathogen virulence by regulating the production of the siderophore pyoverdine. In this study, we investigated the role of PqsA, a key cell-signaling protein, in this regulatory pathway. We demonstrate that PqsA promotes pyoverdine production in a biofilm-dependent manner. Under nutritionally deficient conditions, where biofilm and pyoverdine are decoupled, PqsA is dispensable for pyoverdine production. Interestingly, although PqsA-dependent pyoverdine production does not rely upon Pseudomonas quinolone signal (PQS) biosynthesis, exogenous PQS can also trigger biofilm-independent production of pyoverdine. Adding PQS rapidly induced planktonic cell aggregation. Moreover, these clumps of cells exhibit strong expression of pyoverdine biosynthetic genes and show substantial production of this siderophore. Finally, we surveyed the relationship between biofilm formation and pyoverdine production in various clinical and environmental isolates of *P. aeruginosa* to evaluate the clinical significance of targeting biofilm during infections. Our findings implicate PqsA in *P. aeruginosa* virulence by regulating biofilm formation and pyoverdine production.

## 1. Introduction

Multi-drug resistant, gram-negative bacteria, including *Pseudomonas aeruginosa*, are one of the leading causes of nosocomial infections in intensive care units [1]. It is increasingly common for patients to be exposed to multi- or pan-drug resistant strains, complicating treatment of chronic conditions, resulting in acute, life-threatening infections. Antimicrobial resistant infections are associated with pronounced increases in morbidity and mortality, along with drastically increasing healthcare costs [1,2]. Beyond expressing a wide-variety of resistance genes acquired via horizontal gene transfer, *P. aeruginosa* utilizes two methods to defend against antimicrobial agents. First, during chronic infections, bacteria form dense biofilm structures on the surface of host tissue and at interfaces with medical implants. Bacteria embedded in these structures are virtually impervious to antibiotics and often evade recognition by the host’s innate immune system. Second, bacteria express multi-drug efflux pumps that efficiently reduce intracellular concentration of harmful toxic molecules [3,4,5]. These innate defense mechanisms complicate treating chronic *P. aeruginosa* infections. For example, over half of patients with cystic fibrosis suffer from chronic colonization, and infection is estimated to shorten their life expectancy by nearly ten years [6,7]. In addition, the prevalence of *P. aeruginosa* in nosocomial infections is rising, making it imperative that we search for a new therapeutic approach to support the dwindling identification of new antimicrobials.

Inhibition of biofilm formation represents an appealing target for improving clinical outcomes. Genetic and chemical disruption of biofilm in various model host systems has demonstrated the utility of this approach. For instance, mutations in genes involved in biofilm formation (i.e*.*, Pel exopolysaccharide biosynthesis, flagella assembly, Pseudomonas quinolone signal (PQS) biosynthesis) have proven sufficient to rescue hosts in *Caenorhabditis elegans*, *Drosophila melanogaster*, and murine infection models, indicating that biofilm formation is often required for full *P. aeruginosa* pathogenicity [8,9,10]. Furthermore, chemical disruption of biofilm via novel inhibitors such as *meta*-bromo-thiolactone, 2-aminoamidazole derivatives, zingerone, hydrogenated furanone, or *S*-phenyl-l-cysteine sulfoxide was also sufficient for significant host rescue during pathogenesis [8,11,12,13,14]. To date, most of these compounds limit biofilm formation by targeting *P. aeruginosa* quorum sensing.

However, there are two main challenges to targeting biofilm in *P. aeruginosa*. The first is the complex structural composition of extracellular matrices. Biofilm matrices are generally comprised of four core components: exopolysaccharides, extracellular DNA, flagella/pili, and secreted peptides that provide structural integrity and facilitate surface attachment [15,16,17,18,19]. The relative proportion and nature of these components vary amongst *P. aeruginosa* isolates. For instance, the *P. aeruginosa* reference strain PAO1 secretes three polysaccharides: Pel, alginate (shared by PA14, another commonly used *P. aeruginosa* reference strain), and Psl, which is not produced by PA14 [20]. Further complicating matters, each component is responsible for distinct stages of biofilm formation. Initial bacterial surface attachment typically involves flagella, microcolonies are formed via fimbriae elements, and mature biofilms develop through the production of exopoloysaccharides [21].

The second challenge is the regulatory complexity of the biosynthesis of extracellular polymeric substances. Production of flagella, pili, and exopolysaccharides is controlled by both intracellular signaling via secondary messengers (i.e., cyclic diguanylate monophosphate, c-di-GMP) and quorum sensing via secreted signaling molecules like PQS, C4-HSL (the RhlRI system), and C12-HSL (LasRI system) [22,23,24,25,26,27,28,29]. The global two-component regulator GacA/S regulates the expression of these signaling molecules via the small RNAs RsmY and RsmZ [30]. Despite extensive in vitro studies demonstrating the role of these factors in biofilm formation, it remains difficult to predict the effects of targeting a single signaling pathway during *P. aeruginosa* infection.

Biofilm formation in *P. aeruginosa* has a complex, bidirectional regulatory relationship with the siderophore pyoverdine. Pyoverdine is responsible for obtaining extracellular iron, a nutrient essential for biofilm formation in various species of bacteria including *P. aeruginosa* [31,32,33,34,35]. Previous reports have demonstrated that pyoverdine biosynthesis is necessary for full biofilm formation under iron-starved conditions [33]. We recently demonstrated that the inverse regulation also occurs [8]. Pyoverdine is regulated by biofilm formation when bacteria are not iron-starved. Compromising biofilm development by either genetic or chemical disruption severely decreased pyoverdine biosynthesis and rescued *C. elegans* from *P. aeruginosa* pathogenesis. Due to its roles in iron acquisition, regulation of secreted toxins such as the translational inhibitor ToxA and the protease PrpL, disruption of host mitochondrial function, and observations that compromising pyoverdine production is sufficient to rescue hosts from infection, pyoverdine is an important therapeutic target [36,37,38,39,40,41].

In this study, we investigate the effects of genetic disruption of PQS biosynthesis genes and exogenous PQS on *P. aeruginosa* virulence by observing its effects on biofilm and pyoverdine. Our results demonstrate that the PQS biosynthetic protein PqsA was indispensable for biofilm formation and, subsequently, pyoverdine production. However, this phenomenon was specific to PqsA and was not dependent on the biosynthesis of PQS, as a *pqsH* mutant did not display compromised pyoverdine production. Exogenous PQS induced autoaggregation of *P. aeruginosa* cells, even in the absence of flagella or Pel exopolysaccharide. These cell aggregates showed high levels of pyoverdine biosynthetic gene expression, promoting pyoverdine production in a biofilm-independent manner.

## 2. Results

### 2.1. Exogenous PQS Induces Autoaggregation of Planktonic Cells and Enhances Pyoverdine Production in a Biofilm-Independent Manner

**Figure 1:**
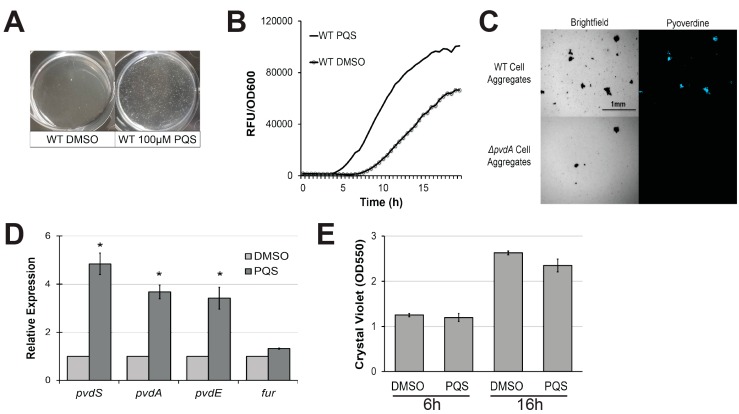
Exogenous Pseudomonas quinolone signal (PQS) induces cell aggregation and promotes pyoverdine production. (**A**) Cell aggregate formation in *P. aeruginosa* PA14 treated with either dimethyl sulfoxide (DMSO) (left) or 100 µM PQS (right) after a 4 h growth period. (**B**) Pyoverdine fluorescence normalized to bacterial growth, measured over 24 h in *P. aeruginosa* treated with DMSO or 100 µM PQS. (**C**) Brightfield (left) or fluorescence (right) micrographs of pyoverdine expression in either wild-type PA14 (top) or PA14*ΔpvdA*, a pyoverdine biosynthesis mutant (bottom). Cell aggregates were visualized with a pyoverdine-specific fluorescence filter. (**D**) Expression of pyoverdine biosynthesis genes in bacteria treated with 100 µM PQS or DMSO after 6 h growth, as measured by quantitative, real-time PCR (qRT-PCR). Gene expression in PQS-treated bacteria was normalized to the solvent control. (**E**) Quantification of crystal violet staining of biofilm matrix from wild-type bacteria treated with either DMSO solvent or 100 µM PQS after 6 or 16 h growth. Crystal violet was solubilized in 30% acetic acid solution and quantified by absorbance at 550 nm. Error bars in (**D**) represent standard error of the mean (SEM) between three biological replicates. * Corresponds to *p* < 0.01 based on Student’s *t*-test. Pyoverdine production curves without normalization to bacterial growth are available in Figure S1.

2-Heptyl-3,4-dihydroxyquinoline, also known as Pseudomonas quinolone signal or PQS, is a small quorum-sensing molecule produced by *P. aeruginosa.* PQS is involved in the regulation of a wide variety of phenomena in the pathogen, including iron homeostasis and the production of multiple virulence factors [42]. Since extracellular PQS largely functions as a quorum-sensing molecule, we tested its impact on pyoverdine production by supplementing growth medium with commercially-sourced, purified PQS. Within 4 h, we observed aggregation and sedimentation of planktonic cells (Figure 1A), which resulted in significantly earlier activation of pyoverdine biosynthesis, as demonstrated by measuring pyoverdine’s inherent fluorescence (Figure 1B). This is consistent with previous reports of a link between pyoverdine and cell aggregation [43]. *P. aeruginosa* treated with exogenous 2-aminoacetophenone (2-AA), another secreted molecule produced from 2-aminobenzoylacetate (2-ABA), the precursor of PQS, neither triggerd cell aggregation nor exhibited changes in pyoverdine kinetics (Figure S2A–C) [44]. We used fluorescence microscopy to visualize pyoverdine expression and observed that aggregated planktonic cells, which congregated after PQS treatment, exhibited high levels of pyoverdine production, as shown by increased pyoverdine-specific fluorescence (Figure 1C). When a pyoverdine-deficient mutant, *P. aeruginosa* PA14*ΔpvdA,* was treated with PQS, cell aggregation still occurred, but fluorescence was abolished, verifying that the fluorescence observed was from pyoverdine. We also measured the expression of pyoverdine biosynthesis genes in PQS-induced cell aggregates by quantitative, real-time PCR (qRT-PCR). *pvdS*, *pvdA*, and *pvdE* (genes encoding an alternate sigma factor responsible for the expression of most pyoverdine biosynthetic genes, biosynthesis of pyoverdine precursors, and pyoverdine transport, respectively) were expressed more highly in PQS-treated *P. aeruginosa* cells (Figure 1D). This was not due to changes in expression of the ferric uptake regulator gene (*fur*) (Figure 1D). This is consistent with previous findings by Visaggio and colleagues, who observed that artificial cell aggregation restored pyoverdine production in exopolysaccharide-deficient *P. aeruginosa* mutants [43]. PQS supplementation did not increase biofilm production at early (when pyoverdine production was initiated) or later timepoints (Figure 1E). Combined, these findings suggest that PQS, when exogenously added to bacteria, can induce pyoverdine production in a biofilm-independent manner, likely by inducing aggregation of planktonic cells. 

**Figure 2:**
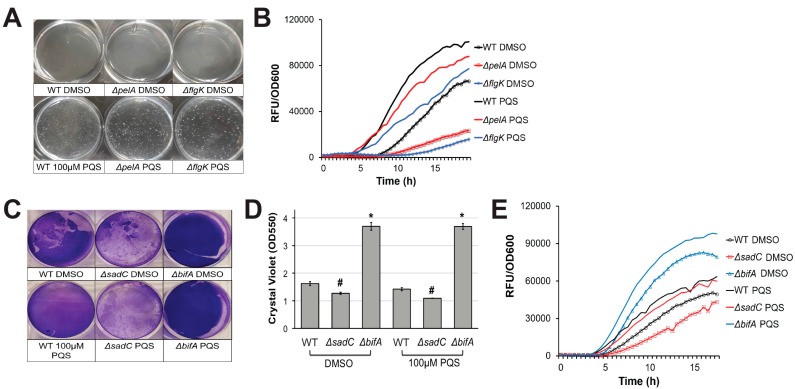
Exogenous PQS restores pyoverdine kinetics in biofilm-defective mutants. (**A**) Cell aggregate formation in wild-type PA14 or biofilm mutants treated with either DMSO (top) or 100 µM PQS (bottom) after 4 h growth. (**B**) Pyoverdine fluorescence normalized to bacterial growth measured over 24 h in biofilm mutants treated with DMSO or 100 µM PQS. (**C**) Biofilm matrix of wild-type (WT) PA14 or c-di-GMP biosynthetic mutants grown in the presence of 100 µM PQS or DMSO solvent for 24 h. Biofilm matrices were stained with 0.1% crystal violet. (**D**) Quantification of crystal violet stain measured by absorbance at 550 nm after solubilizing in 30% acetic acid solution. (**E**) Pyoverdine fluorescence, normalized to bacterial growth, measured kinetically over 24 h in c-di-GMP biosynthetic mutants grown in the presence of 100 µM PQS or DMSO solvent. Error bars in (**D**) represent SEM between three biological replicates. NS corresponds to *p* > 0.05, # corresponds to *p* < 0.05, and * corresponds to *p* < 0.01 based on Student’s *t*-test. Pyoverdine production curves without normalization to bacterial growth are available in Figure S1.

In a previous report, we noted that *P. aeruginosa* cells that had aggregated and were initiating biofilm development showed high levels of pyoverdine fluorescence [8]. *P. aeruginosa* mutants defective in exopolysaccharide, Type IV pili, or flagellar biosynthesis (which are all components of biofilm formation) failed to properly form these aggregates in the biofilm matrix and also had impaired pyoverdine production [8]. We hypothesized that the absence of these aggregates disrupted the initiation of pyoverdine signaling in the system, preventing the pathogen from amplifying pyoverdine production beyond its basal level. In addition to its iron-scavenging activity, pyoverdine also functions as a signaling molecule; when iron-bound pyoverdine binds to the ferripyoverdine receptor protein FpvA in the cell membrane, PvdS is released from its sequestration by FpvR, activating transcription of pyoverdine biosynthesis genes, including *pvdS* [38,45,46]. This auto-upregulatory mechanism is likely impaired in biofilm mutants. Since extracellular PQS induces cell autoaggregation, we hypothesized that these cell-cell interactions may be sufficient to induce pyoverdine production, even in the absence of the normally required biofilm signal. As expected, pyoverdine production in a *ΔpelA* expolysaccharide biosynthetic mutant was restored by exogenous PQS (Figure 2A,B). In a PA14 *ΔflgK* flagellum biosynthetic mutant, the lag before pyoverdine production was significantly shortened after PQS supplementation (Figure 2A,B). Similar results were observed when *P. aeruginosa* c-di-GMP biosynthetic mutants were grown in the presence of exogenous PQS. We previously reported that intracellular c-di-GMP positively regulates pyoverdine production in a biofilm-dependent manner [8]. PQS also restored pyoverdine production in a diguanylate cyclase mutant, PA14*ΔsadC*, which exhibits poor biofilm formation and pyoverdine production (Figure 2C–E) [8]. Due to its hyperbiofilm phenotype, a phosphodiesterase mutant, PA14*ΔbifA*, exhibits early activation of pyoverdine production, even in the absence of exogenous PQS (Figure 2C–E) [8]. The addition of PQS further enhanced pyoverdine production in this mutant (Figure 2C–E). These observations support our conclusion that exogenous PQS promotes pyoverdine in a biofilm-independent manner. Furthermore, although PQS treatment largely restored pyoverdine production in biofilm mutants, it was still significantly lower than PQS-supplemented wild-type cells. This suggests that biofilm also has a PQS-independent impact on pyoverdine production. 

### 2.2. PqsA Regulates Pyoverdine in a Biofilm-Dependent Manner

**Figure 3:**
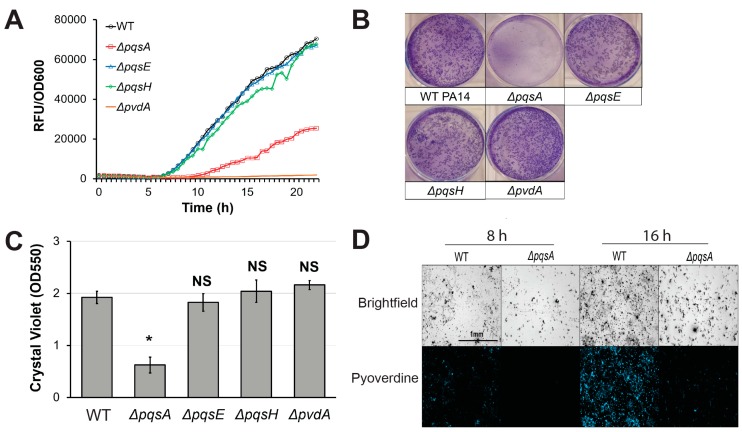
PqsA is necessary for biofilm formation and pyoverdine production. (**A**) Pyoverdine fluorescence, normalized to bacterial growth, measured kinetically over 24 h in WT PA14 and PQS biosynthetic mutants. (**B**) Biofilm matrix of PQS biosynthetic mutants in 6-well plate stained with 0.1% crystal violet. (**C**) Quantification of crystal violet stain measured by absorbance at 550 nm after solubilizing in 30% acetic acid solution. (**D**) Brightfield (top) and fluorescence (bottom) micrographs of WT PA14 and *ΔpqsA* biofilm matrix cell aggregates visualized with a pyoverdine-specific fluorescence filter. Error bars in (**B**) represent SEM between three biological replicates. NS corresponds to *p* > 0.05, # corresponds to *p* < 0.05, and * corresponds to *p* < 0.01 based on Student’s *t*-test. Pyoverdine production curves without normalization to bacterial growth are available in Figure S1.

Next, we investigated the effects of genetic disruption of PQS biosynthesis on pyoverdine production. PA14*ΔpqsA*, a PQS biosynthetic mutant, exhibited impaired pyoverdine production (Figure 3A). Surprisingly however, this phenomenon was not observed in *pqsE* or *pqsH* mutants, suggesting that PqsA plays a unique role in pyoverdine regulation independently of PQS biosynthesis. Previous studies demonstrated that PqsA is indispensable for proper biofilm formation in *P. aeruginosa* [47,48,49]. Consistent with these reports, PA14*ΔpqsA* exhibited poor biofilm formation in static M9 media growth conditions (Figure 3B,C) [47,48,49]. Deletion of *pqsE* or *pqsH* did not significantly affect biofilm formation, indicating that the observed disruption of biofilm in PA14*ΔpqsA* was not due to impaired PQS synthesis. PA14*ΔpqsA* mutant also produced fewer fluorescent clusters of aggregated cells in the biofilm matrix than wild-type PA14 at both early and late time points (Figure 3D). This phenomenon has been previously observed in *P. aeruginosa* biofilm mutants [8]. These results are consistent with our previous model, where biofilm formation was necessary for pyoverdine production [8]. According to this model, *P. aeruginosa* mutants that cannot properly initiate biofilm lack the cell aggregates that express high levels of pyoverdine, nucleating siderophore production. 

Experiments investigating the role of PqsA in another *P. aeruginosa* reference strain, PAO1, reflected the complexity of biofilm regulation. Interestingly, although *pqsA* disruption still attenuated biofilm formation and pyoverdine production, the decrease was much more modest (Figure S3A,B). These data suggest that PAO1 may utilize additional pathways to control biofilm formation and pyoverdine production. These data correlate with our observations that PAO1 produces more biofilm and pyoverdine than PA14 and that the lag preceding pyoverdine production is shorter [8]. Overall, this suggests that the regulation of biofilm formation varies between *P. aeruginosa* strains.

**Figure 4:**
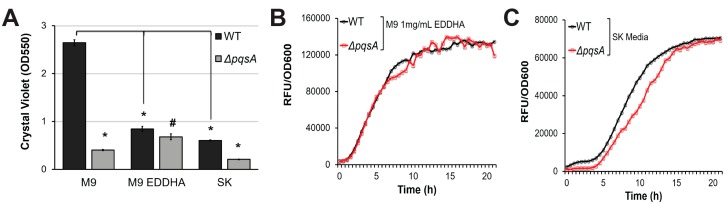
PqsA does not regulate pyoverdine production in low-nutrient conditions. (**A**) Quantification of crystal violet staining of biofilm matrix from wild-type PA14 or PA14*ΔpqsA* grown in M9 media, M9 media supplemented with 1 mg/mL ethylenediamine N,N′-bis(2-hydroxyphenylacetic acid) (EDDHA), or SK media. Crystal violet was solubilized in 30% acetic acid solution and quantified by absorbance at 550 nm. (**B**,**C**) Kinetic measurements of pyoverdine fluorescence, normalized to bacterial growth, in wild-type PA14 or PA14*ΔpqsA* grown in (**B**) M9 supplemented with 1mg/mL EDDHA or in (**C**) SK media. Error bars in (**A**) represent SEM between three biological replicates. # corresponds to *p* < 0.05, and * corresponds to *p* < 0.01 based on Student’s *t*-test. Pyoverdine production curves without normalization to bacterial growth are available in Figure S1.

To validate the hypothesis that PqsA regulates pyoverdine via biofilm, we took advantage of our prior demonstration that biofilm and pyoverdine can be unlinked by limiting nutritional quality of the media [8]. Limiting iron, which is necessary for proper biofilm formation, is a convenient means to accomplish this separation [31,32,33]. *P. aeruginosa* grown in the presence of the strong, non-utilizable iron chelator ethylenediamine N,N′-bis(2-hydroxyphenylacetic acid) (EDDHA) produces pyoverdine, despite poor biofilm formation and limited bacterial growth [8]. Similar results were observed when bacteria were grown in nutrient-poor slow-kill (SK) media used for *C. elegans* Liquid Killing [8,50]. Under these growth conditions, *P. aeruginosa* exhibited severely impaired biofilm formation (Figure 4A and [8]). Previously, we reported that under these conditions mutants with impaired biofilm formation (PA14*∆pilY1*, PA14*∆pelA*, PA14*∆flgK*) demonstrate wild-type levels of pyoverdine production, despite their biofilm formation being even lower than that of wild-type. Consistent with biofilm mutants, *pqsA* mutant exhibited attenuation of biofilm compared to wild-type bacteria under nutrient-poor conditions, even though the difference was much less dramatic (Figure 4A). However, its pyoverdine production kinetics were undisrupted, suggesting that the regulatory link between PqsA, pyoverdine, and biofilm does not exist under conditions of limited biofilm production (Figure 4B,C). This observation, combined with our previous conclusion that genetic disruption of biofilm does not affect pyoverdine production in the presence of EDDHA or SK media [8], suggest that PqsA regulates pyoverdine production via biofilm formation only under high-nutrient, high-biofilm conditions.

### 2.3. Pyoverdine Production Correlates with Biofilm-Forming Capacity in a Subset of P. aeruginosa Isolates

**Figure 5:**
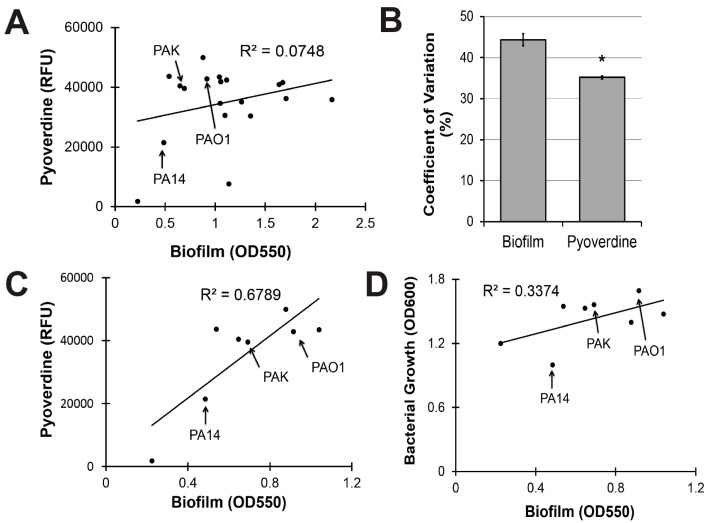
Pyoverdine production correlates with biofilm formation, but only in *P. aeruginosa* strains with limited biofilm-forming capacity. (**A**) Correlation between biofilm formation and pyoverdine production in 19 clinical and environmental isolates of *P. aeruginosa* after 24 h growth. Biofilm formation was measured by concentration of dissolved crystal violet biofilm stain (OD550). Pyoverdine was measured spectrophotometrically. (**B**) Coefficient of variation for biofilm formation and pyoverdine production across 19 *P. aeruginosa* isolates (standard deviation/mean × 100). (**C**) Correlation between biofilm formation and pyoverdine production in 8 isolates with low biofilm formation. (**D**) Correlation between bacterial growth (measured by absorbance at 600 nm) and biofilm formation in 8 isolates with low biofilm formation. Error bars in (**B**) represent SEM between three biological replicates. * Corresponds to *p* < 0.01 based on Student’s *t*-test. Raw data from scatterplots are available in Table S1.

To evaluate the clinical utility of targeting biofilm production to mitigate pyoverdine-mediated virulence, we investigated the relationship between biofilm formation and pyoverdine production in 18 additional clinical and environmental isolates of *P. aeruginosa*. These strains were isolated from multiple sources, including infection sites that ranged from the lung of a cystic fibrosis patient to urinary tract, blood, and ocular infections [51]. Although we previously demonstrated that biofilm formation correlated with pyoverdine production in two reference strains of *P. aeruginosa* (PA14 and PAO1), this trend did not hold for the panel as a whole (Figure 5A). 

Statistical analysis indicated that biofilm production was more variable than pyoverdine secretion across this panel of *P. aeruginosa* isolates. We calculated the coefficient of variation for biofilm formation and pyoverdine production as a means to quantify the variability. Biofilm had a 44% variation amongst strains while the variation for pyoverdine was only 35%, suggesting that biofilm formation is more variable (Figure 5B).

When our analysis was limited to the eight isolates that made the least biofilm, a much stronger, positive correlation was observed (Figure 5C). While bacterial growth explained a portion of this correlation (Figure 5D, all raw data available in Table S1), significant residual remained. Interestingly, the three strains most commonly used as reference strains for *P. aeruginosa* (PAO1, PA14, and PAK) were all in the low biofilm subset (Figure 5C). Within this subset, there is some evidence that compromising PqsA inhibits biofilm and pyoverdine. Beyond a certain threshold, however, changes in biofilm density cease impacting pyoverdine production, suggesting that the regulatory mechanism has reached saturation and that PqsA is unlikely to be a useful target for limiting pyoverdine production in these strains.

## 3. Discussion

**Figure 6:**
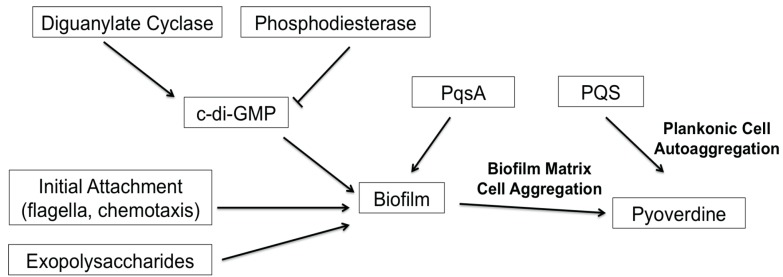
Model for biofilm-mediated regulation of pyoverdine. This model summarizes factors involved in biofilm formation that have been shown to affect pyoverdine production.

Despite the increasing desire to target biofilm formation, the development of biofilm inhibitors has been slow. In part, this is due to the number of genes and the complexity of the pathways involved. For example, a high-throughput biofilm screen conducted by Musken and colleagues showed that 394 genes (out of approximately 6000) significantly contribute to biofilm formation [47]. Frustratingly, many of these genes have yet to be characterized. The screen recapitulated many previous findings on the regulation of *P. aeruginosa* biofilms; compromising genes that function in the biosynthesis of extracellular polymeric substances (i.e., exopolysaccharides) or upstream regulatory pathways (i.e., RhlRI quorum sensing system, c-di-GMP biosynthesis) affected biofilm formation [16,22,25]. Complicating things further, many of these upstream regulators are also associated with other cell-cell signaling and virulence functions in the bacteria, such as type III secretion and pyoverdine production [8,26,52]. In this study, we demonstrated that the PQS biosynthetic protein PqsA regulates pyoverdine production in a biofilm-dependent, but PQS-independent manner. We also identified additional complexities in the *P. aeruginosa* quorum-sensing system; although PQS biosynthesis does not affect pyoverdine production, exogenous PQS promoted pyoverdine production via planktonic cell aggregation. Previously, we hypothesized that cell aggregates embedded in extracellular matrices were the cells that initiated pyoverdine expression, allowing a feed-forward loop that stimulated expression in other cells [8]. In this case, pyoverdine production occurs before biofilm formation, and is likely induced by the aggregation of the cells due to the quorum signal. Reestablishment of pyoverdine biosynthesis in PQS-treated biofilm mutants also supports this model, since PQS can stimulate pyoverdine production in a biofilm-independent manner. Overall, our results support a model wherein cell aggregation, rather than biofilm formation, is necessary and sufficient for pyoverdine production. Based on these findings, we generated a model for biofilm-mediated regulation of pyoverdine (Figure 6) that is consistent with previous publications by multiple labs [8,16,17,28,29,43].

It is important to note that this regulatory mechanism is observed in M9-CAA media static growth conditions where iron is not severely limiting. A previous study by Banin and colleagues demonstrated that pyoverdine biosynthesis is necessary for full biofilm formation under iron-starved conditions [33]. However, under our growth conditions, bacteria were able to properly form biofilm even in the absence of pyoverdine (as observed in PA14*∆pvdA*), suggesting that the bacteria were not iron-starved. Furthermore, when bacteria were treated with EDDHA, essentially depriving them of ferric iron, both biofilm formation and pyoverdine production were predominantly regulated by intracellular iron levels. These results demonstrate that biofilm-mediated regulation of pyoverdine is likely independent of the normal Fur-dependent regulation of pyoverdine production [43]. Overall, these findings suggest the existence of two distinct regulatory pathways between pyoverdine and biofilm that occur under separate growth conditions that vary by environmental iron concentrations. 

Numerous studies have culminated in an exhaustive list of factors involved in biofilm formation. The therapeutic utility of targeting critical nodes of this network remains unclear. It is still uncertain whether compromising any single virulence factor can serve as a “silver bullet” in *P. aeruginosa* infections. Perhaps the most promising approach is to focus on the overall attenuation of pathogen virulence rather than one specific virulence factor. One obvious target is c-di-GMP. Modulating intracellular c-di-GMP has a pronounced effect on biofilm formation through its role in Pel expolysaccharide biosynthesis and flagellar assembly and results in significant disruption of pyoverdine production [8,22]. c-di-GMP levels also regulate the expression of the type III and type VI secretion systems, which are crucial mediators of acute virulence [52]. The safety of targeting c-di-GMP remains an open question, however, since it is involved in many prokaryotic and even some eukaryotic signaling pathways, raising the specter of off-target effects. In this study, we also investigated the therapeutic utility of PQS biosynthesis genes. The disruption of PqsA has the potential to attenuate *P. aeruginosa* virulence in three ways: by limiting PQS quorum-sensing; by limiting biofilm formation, which increases bacterial susceptibility to antimicrobials and immune cells; and by attenuating pyoverdine and its downstream effectors, which also interferes with general iron homeostasis in the pathogen [36,38,53,54]. However, we also observed that these effects may vary amongst various *P. aeruginosa* strains. 

To gain insight into this variation, we conducted a survey of biofilm formation in 19 different *P. aeruginosa* strains (isolated from various clinical and environment sources). Biofilm formation was highly variable across these isolates. Surprisingly, the three most commonly studied *P. aeruginosa* strains, PA14, PAO1, and PAK, produced less biofilm than more than half of the isolates. These results suggest that the application of our current knowledge on biofilm and its inhibition is extremely limited and may apply only to a small subset of *P. aeruginosa* infections. Furthermore, pyoverdine showed a strong, positive correlation to biofilm formation only in the isolates that exhibit relatively poor biofilm formation (including the three reference strains). Interestingly, similar variations have been seen in comparative pathogenicity studies when large panels of *P. aeruginosa* strains were tested in *C. elegans* slow-kill and mouse corneal infection studies. Both models demonstrated varying levels of virulence for different *P. aeruginosa* isolates [55,56]. In *C. elegans*, this ranged from supporting growth and reproduction of the host to outright killing in only a few days; interestingly, the isolates’ virulence were also dependent on the type of assay used [55,57]. Similarly, clinical isolates of *P. aeruginosa* exhibit >1000-fold differences in infectious dose in a mouse corneal infection model [56]. These findings demonstrate the necessity of studying *P. aeruginosa* strains beyond the conventional references to better understand the determinants of pathogenicity. It also highlights the fact that many of these studies are performed in vitro; ultimately, host-pathogen interactions are going to be more complex and may also dramatically impact the results of targeting virulence determinants. 

## 4. Materials and Methods

### 4.1. Strains and Growth Conditions

Bacterial strains are listed in Table 1. For all experiments (unless otherwise mentioned), *P. aeruginosa* was statically grown in M9 media (M9 salts (1% *w*/*v*), casamino acids (1% *w*/*v*), 1 mM MgSO_4_, 1 mM CaCl_2_) in 6-well plates (Greiner, Monroe, NC, USA) at 30 °C. SK media was composed of 0.35% (*w*/*v*) Bacto-Peptone, 0.3% (*w*/*v*) NaCl, 1 mM MgSO_4_, and 1 mM CaCl_2_ [58].

### 4.2. Biofilm Formation Assay

Detailed procedure is available in [8]. In brief, overnight bacterial cultures grown in Luria Broth (LB) were diluted 20-fold into M9 media in 6-well plates (2 mL/well) (Greiner, NC, USA). Bacteria were grown statically for 24 h at 30 °C. Biofilms were stained with 0.1% (*w*/*v*) crystal violet in 20% (*v*/*v*) ethanol/water. Biofilm stain was quantified by dissolving crystal violet in 30% (*v*/*v*) acetic acid solution and measuring absorbance at 550 nm using a Cytation5 multimode reader (BioTek, Winooski, VT, USA).

### 4.3. Pyoverdine Production Kinetics

*P. aeruginosa* static cultures were prepared as described above. The plate was incubated inside a Cytation5 multimode reader (BioTek, Winooski, VT, USA) under constant temperature control at 30 °C. Pyoverdine fluorescence (Ex. 405, Em. 460) and bacterial density (absorbance at 600 nm) measurements were made every 30 min.

### 4.4. Pyoverdine Fluorescence Microscopy

Detailed procedure and fluorescence filter specifications are available in [8]. To image biofilm matrices, after 8 or 16 h of bacterial growth in static 6-well plate cultures media and planktonic cells were aspirated from the plate. The biofilm matrix was washed with 2 mL PBS buffer (Gibco, Waltham, MA, USA) and imaged in a Cytation5 multimode plate reader ( BioTek, Winooski, VT, USA) using a custom pyoverdine-specific fluorescence filter. All images were taken under identical conditions. To image planktonic cell aggregates, after 6 h of bacterial growth media with planktonic cell aggregates were collected from the plate. Cells were washed and resuspended in 1mL PBS buffer (Gibco, Waltham, MA, USA). Resuspended cells were transferred to a new 6-well plate for imaging.

### 4.5. RNA Purification and qRT-PCR

After 6 h growth in 6-well plates, planktonic cell aggregates were collected from 1.5 mL of supernatant, gently washed, and RNA was extracted using Trizol reagent (Invitrogen, Carlsbad, CA, USA). Reverse transcription was performed using random decamers and Retroscript Kit (Ambion, Waltham, MA, USA). qRT-PCR was conducted in a CFX-96 real-time thermocycler (Bio-Rad, Hercules, CA, USA) using PerfeCTa SYBR Green Fastmix (Quantabio, Beverly, MA, USA) as fluorescent nucleic acid dye. Fold changes were calculated using a ΔΔ*C*_t_ method with *proC* expression as reference gene control. Gene expression in bacteria treated with PQS was normalized to that of DMSO solvent control. Primer sequences are available upon request.

### 4.6. Statistical Analysis

Statistical significance was evaluated using two-sample unpaired *t*-test analysis.

## Figures and Tables

**Table 1:** Bacterial strains used in this study.

Bacterial Strains	Relevant Information	Source
*P. aeruginosa* PA14 Strains		
*P. aeruginosa* PA14	WT	[59]
PA14*ΔpqsA*	Secondary metabolites mutant	
PA14*ΔpqsE*	Secondary metabolites mutant	[60]
PA14*ΔpqsH*	PQS biosynthesis mutant	[61]
PA14*ΔpvdA*	Pyoverdine biosynthesis mutant	[62]
PA14*ΔpelA*	Exopolysaccharide deficient biofilm mutant	[28]
PA14*ΔflgK*	Flagella deficient biofilm mutant	[62]
PA14*ΔsadC*	Diguanylate cyclase mutant	[29]
PA14 *ΔbifA*	Phosphodiesterase mutant	[28]
*P. aeruginosa* PAO1 Strains		
*P. aeruginosa* PAO1	WT	[63]
PAO1*pqsA*	Transposon mutant Tc^R^	[64]
*P. aeruginosa* Isolates		
*P. aeruginosa* PAK	Reference Strain	[51]
*P. aeruginosa* CF18	Cystic Fibrosis Isolate	[51]
*P. aeruginosa* CF27	Cystic Fibrosis Isolate	[51]
*P. aeruginosa* CF127	Cystic Fibrosis Isolate	[51]
*P. aeruginosa* E2	Environmental Isolate	[51]
*P. aeruginosa* JJ692	Urinary Tract Infection Isolate	[51]
*P. aeruginosa* MSH3	Environmental Isolate	[51]
*P. aeruginosa* MSH10	Environmental Isolate	[51]
*P. aeruginosa* S35004	Blood Infection Isolate	[51]
*P. aeruginosa* S54485	Urinary Tract Infection Isolate	[51]
*P. aeruginosa* U2504	Urinary Tract Infection Isolate	[51]
*P. aeruginosa* UDL	Urinary Tract Infection Isolate	[51]
*P. aeruginosa* X13273	Blood Infection Isolate	[51]
*P. aeruginosa* X25409	Urinary Tract Infection Isolate	[51]
*P. aeruginosa* 62	Environmental Isolate	[51]
*P. aeruginosa* 6077	Ocular Infection Isolate	[51]
*P. aeruginosa* 19660	Ocular Infection Isolate	[51]

Tc^R^: Tetracycline resistant.

## Supplementary Materials

The following are available online at www.mdpi.com/2076-0817/7/1/3/s1, Figure S1: Pyoverdine production curves without normalization to bacterial growth, Figure S2: Exogenous 2-AA does not promote pyoverdine production, Figure S3: Disruption of PQS biosynthesis in *P. aeruginosa* PAO1 significantly attenuates biofilm formation and pyoverdine production. Table S1: Raw data for Figure 5.
