# Supplementary material for: PqsA Promotes Pyoverdine Production via Biofilm Formation
Source: Pathogens. 2017 Dec 25;7(1):3. doi: 10.3390/pathogens7010003 (PMC5874729; doi:10.3390/pathogens7010003)
Supplement: Supplementary file 1 [file pathogens-07-00003-s001.pdf]

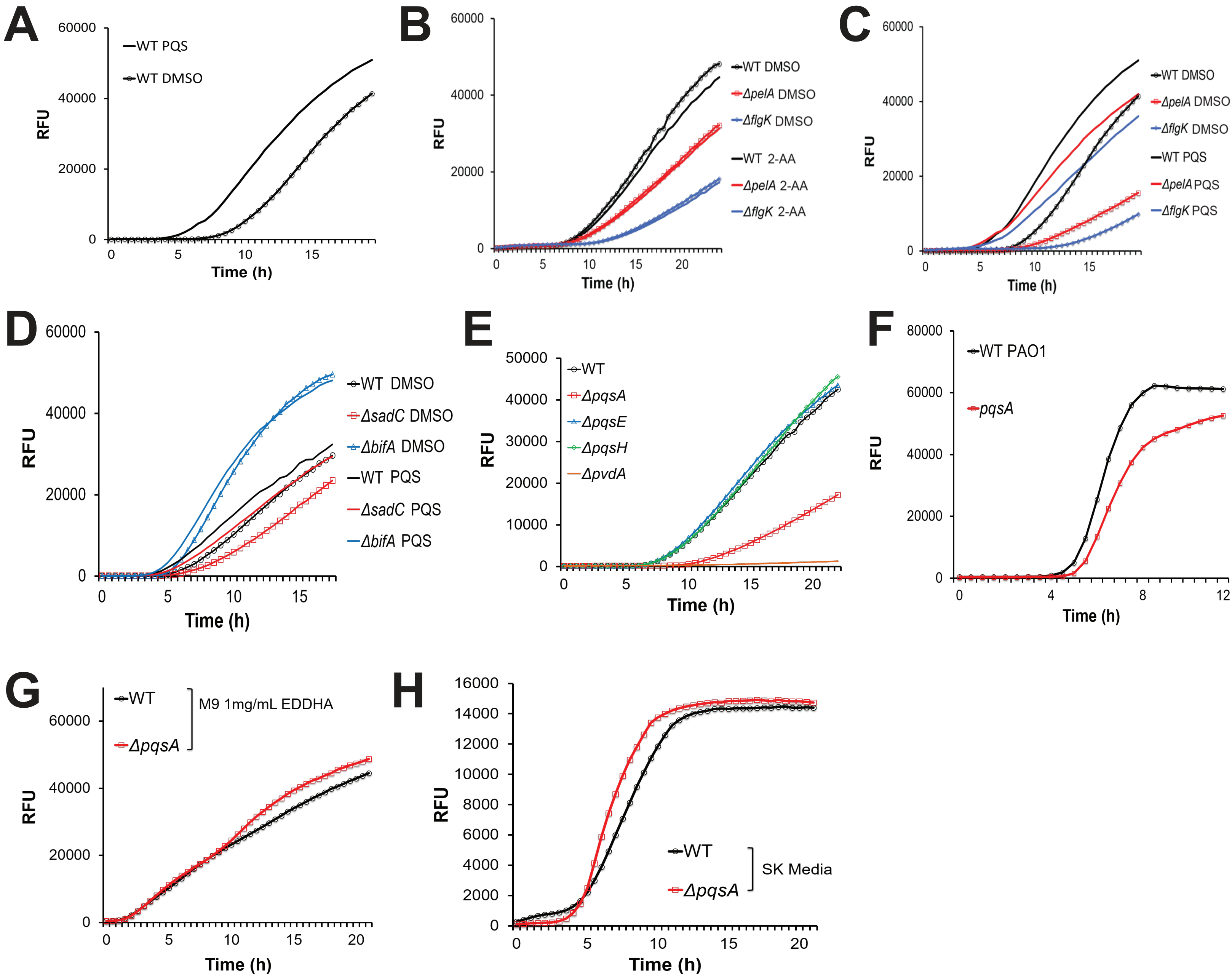

Figure S1. Pyoverdine production curves without normalization to bacterial growth. (A) corresponds to **Figure 1B**. (B) corresponds to **Figure S2C**. (C) corresponds to **Figure 2B**. (D) corresponds to **Figure 2E**. (E) corresponds to **Figure 3A**. (F) corresponds to **Figure S3B**. (G) corresponds to **Figure 4B**. (H) corresponds to **Figure 4C**.

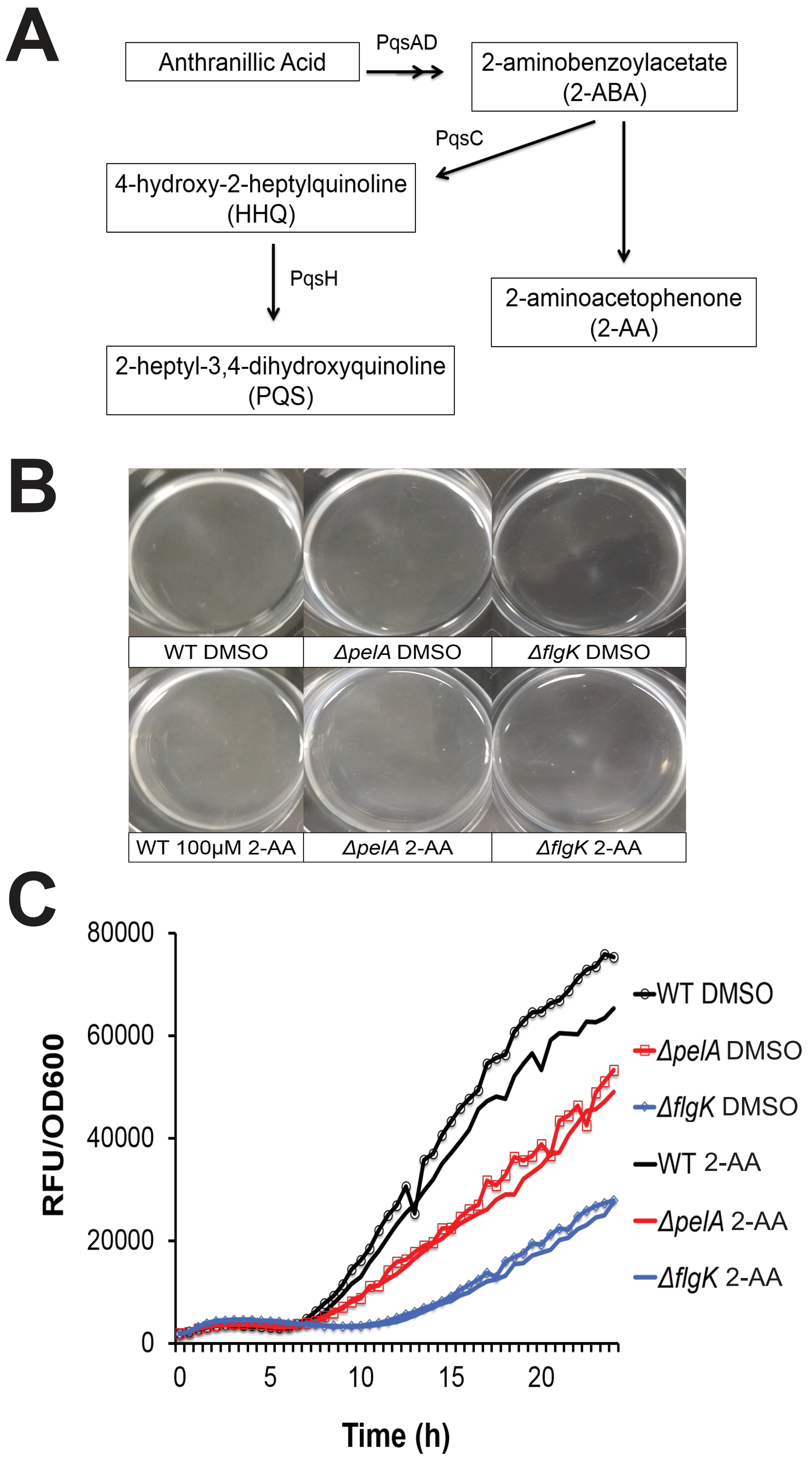

Figure S2. Exogenous 2-AA does not promote pyoverdine production. (**A**) A scheme of PQS and 2-AA biosynthetic pathway [39]. (**B**) The absence of cell aggregate formation in WT PA14 and biofilm mutants treated with 100μM 2-AA after 4 h growth. (**C**) Pyoverdine production normalized to bacterial growth in biofilm mutants treated with 100μM 2-AA measured over 24 h. Pyoverdine production curves without normalization to bacterial growth are available in **Figure S1.**

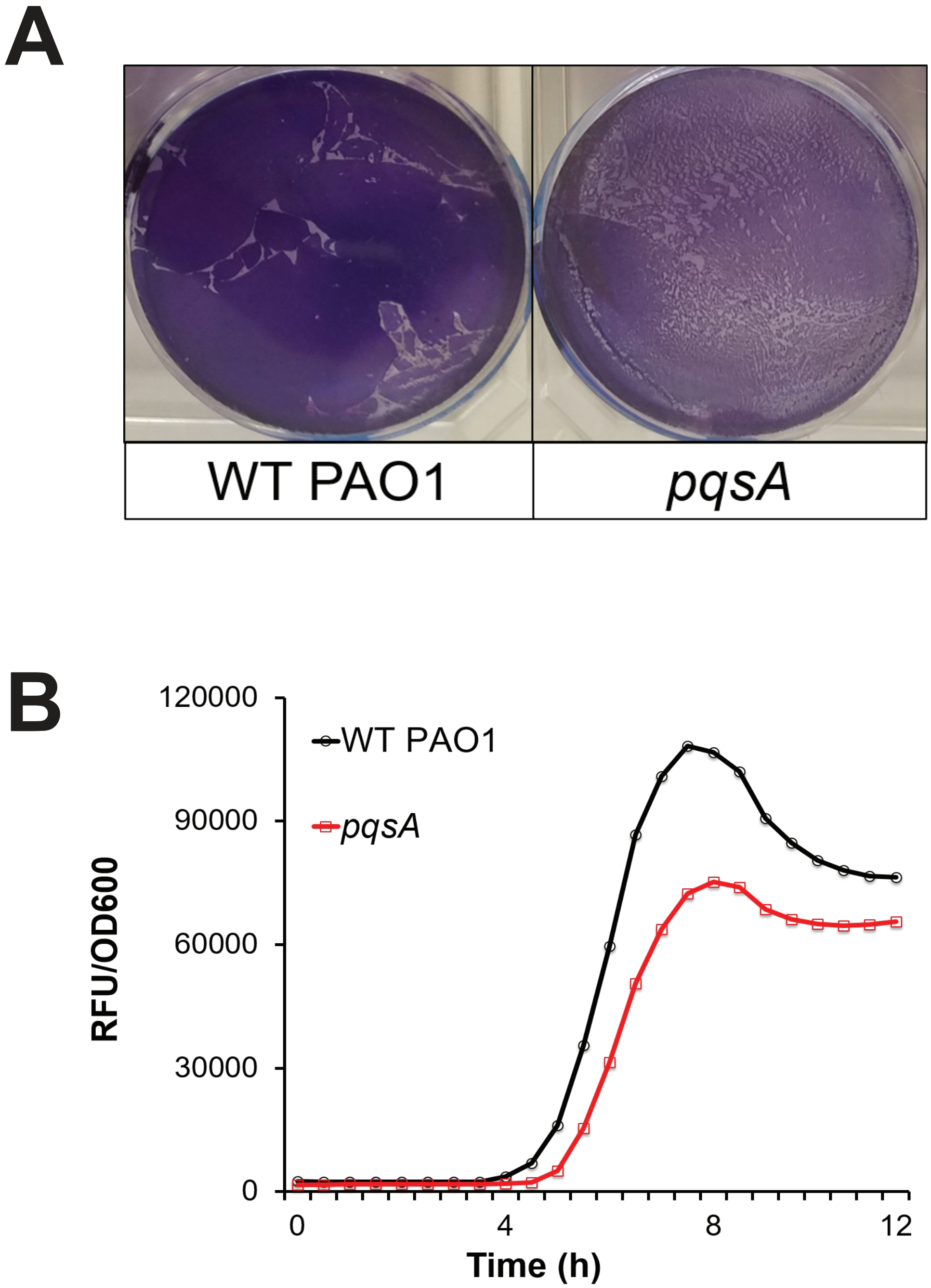

Figure S3. Disruption of PQS biosynthesis in *P. aeruginosa* PAO1 significantly attenuates biofilm formation and pyoverdine production. (**A**) Biofilm matrix of WT PAO1 and a PAO1*pqsA* transposon mutant in 6-well plate stained with 0.1% crystal violet. (**B**) Pyoverdine fluorescence normalized to bacterial growth measured kinetically over 24 h. Pyoverdine production curves without normalization to bacterial growth are available in **Figure S1**.

Table S1. Raw data for **Figure 5**

Isolates	Biofilm (OD550)	Pyoverdine (RFU)	Growth (OD600)
CF27	0.224	1840	1.201
PA14	0.484	21521	1
X13273	0.538	43720	1.55
X25409	0.646	40487	1.531
PAK	0.691	39651	1.564
U2504	0.877	50028	1.398
PAO1	0.915	42899	1.695
19660	1.039	43489	1.475
6077	1.048	34638	0.63
S35004	1.054	41907	1.014
MSH10	1.095	30622	1.706
62	1.111	42487	1.194
UDL	1.135	7653	1.269
CF127	1.263	35132	0.196
JJ692	1.351	30415	0.676
S54485	1.636	40989	0.594
E2	1.673	41679	1.688
MSH3	1.706	36249	1.701
CF18	2.163	35860	0.197
